# Genome-Wide Gene–Environment Interaction Analysis Identifies Novel Candidate Variants for Growth Traits in Beef Cattle

**DOI:** 10.3390/ani14111695

**Published:** 2024-06-05

**Authors:** Tianyu Deng, Keanning Li, Lili Du, Mang Liang, Li Qian, Qingqing Xue, Shiyuan Qiu, Lingyang Xu, Lupei Zhang, Xue Gao, Xianyong Lan, Junya Li, Huijiang Gao

**Affiliations:** 1 Institute of Animal Sciences, Chinese Academy of Agricultural Sciences, Beijing 100193, China; dty9527@126.com (T.D.); likeanning@163.com (K.L.); dulili1996@126.com (L.D.); liangmang87@163.com (M.L.); pbli0201@163.com (L.Q.); xqq18292947845@126.com (Q.X.); qsy527518520@foxmail.com (S.Q.); xulingyang@163.com (L.X.); zhanglupei@caas.cn (L.Z.); gaoxue76@126.com (X.G.); 2 Shaanxi Key Laboratory of Molecular Biology for Agriculture, College of Animal Science and Technology, Northwest A&F University, Yangling, Xianyang 712100, China; lanxianyong79@nwsuaf.edu.cn

**Keywords:** genotype-by-environment interaction, robust estimator, cattle, gene-based analysis, gene-set analysis

## Abstract

**Simple Summary:**

Growth traits have been widely studied as economically important traits in the beef cattle industry. However, traditional studies often miss how these traits change under different environmental conditions, only focusing on single genetic changes that affect traits directly. In our study, we analyzed how genetics and environment interact to affect growth in beef cattle, considering four growth traits and two environmental factors. This analysis uncovered several genetic markers for growth traits that are not usually evident in standard studies, showing that some genes have effects that can be obliterated by environmental conditions Further testing showed whether these genetic markers are grouped in specific genes or functional pathways, helping us understand how genetics can influence growth under different environmental conditions. By uncovering novel genetic loci, genes, and candidate biological mechanisms associated with growth traits, our study provides valuable information for selection prediction and breeding decisions in the beef cattle industry.

**Abstract:**

Complex traits are widely considered to be the result of a compound regulation of genes, environmental factors, and genotype-by-environment interaction (G × E). The inclusion of G × E in genome-wide association analyses is essential to understand animal environmental adaptations and improve the efficiency of breeding decisions. Here, we systematically investigated the G × E of growth traits (including weaning weight, yearling weight, 18-month body weight, and 24-month body weight) with environmental factors (farm and temperature) using genome-wide genotype-by-environment interaction association studies (GWEIS) with a dataset of 1350 cattle. We validated the robust estimator’s effectiveness in GWEIS and detected 29 independent interacting SNPs with a significance threshold of 1.67 × 10^−6^, indicating that these SNPs, which do not show main effects in traditional genome-wide association studies (GWAS), may have non-additive effects across genotypes but are obliterated by environmental means. The gene-based analysis using MAGMA identified three genes that overlapped with the GEWIS results exhibiting G × E, namely *SMAD2*, *PALMD*, and *MECOM*. Further, the results of functional exploration in gene-set analysis revealed the bio-mechanisms of how cattle growth responds to environmental changes, such as mitotic or cytokinesis, fatty acid β-oxidation, neurotransmitter activity, gap junction, and keratan sulfate degradation. This study not only reveals novel genetic loci and underlying mechanisms influencing growth traits but also transforms our understanding of environmental adaptation in beef cattle, thereby paving the way for more targeted and efficient breeding strategies.

## 1. Introduction

Growth traits are frequently recorded and used as important selection criteria in modern beef cattle farm production systems and breeding programs, primarily because they are closely linked to increasing overall beef production, reproduction, and other crucial economic traits, thus affecting profitability [1,2]. However, considering the strong environmental adaptability and widespread distribution of beef cattle, joint genetic evaluations between farms located in different geographic regions and candidate gene localization for growth traits are often extremely unstable and poorly replicable [3,4,5], partly because complex traits are thought to be regulated by a combination of genes, environment, and often overlooked gene–environment interactions (G × E). Thus, the exploitation of gene-environment also known as genotype-by-environment interactions may be an attractive and meaningful approach for identifying novel candidate genes associated with growth traits.

From a biological perspective, G × E can be considered a process by which genetic factors regulate environmental influences and vice versa [6]. In most cases, G × E has been used in the etiology of human psychiatric phenotypes to speculate on pathogenic causes, as it explains why, under specific environmental risk exposure, some individuals develop psychiatric symptoms while others do not [7,8,9]. In contrast, the presence and size of G × E in plants and animals were previously explained by assessing genetic parameters through methods such as reaction norm models [10,11,12], as there was not enough genomic data to support molecular-level exploration until advancement in the development of sequencing technologies reduced the limitations of G × E in genome prediction and genome-wide association analysis in plants and animals [13,14,15].

Genome-wide association studies (GWAS) are powerful in locating candidate genetic loci associated with traits of interest [4,16]. However, typical GWAS were designed to detect only single main effect SNPs that directly affect the phenotype, ignoring potentially useful information from available environmental exposure data since environmental variables were treated as random or systematic effects. Several methods have been invented to overcome this problem, some such as multi-trait models and meta-analysis reducing the burden of multiple testing by pre-selecting variants or integrating statistics from GWASs in different environments [17,18,19], while these approaches may yield more significant SNPs, individual SNP effects are unlikely to provide insight into the higher-order biological mechanisms underlying G × E, and the lack of genome-wide G × E data restricts follow-up analyses such as gene-set analysis, which could elucidate the function of G × E effects [20,21]. Moreover, since SNPs with G × E may not show strong main effects, these approaches may also lead to obscuring potential key interactions [20,22]. Other methods such as single-step GWAS or Bayesian regression model can study global G × E effects across the genome by modeling interactions with polygenic risk scores constructed using SNP main effects from GWAS or estimating the proportion of variance explained by G × E effects [13,23,24], although these methods can detect the presence of G × E, they cannot confirm which SNPs or genes were driving G × E interactions. In addition, the above methods may be limited by the selection of environmental variables or inflated estimates due to heteroskedasticity residuals, etc. [25,26]. We, therefore, chose the genome-wide genotype-by-environment interaction studies (GWEIS) method, which has been proven effective in G × E studies [27,28,29], to study G × E associated with growth traits in beef cattle and to control for error inflation using robust standard errors [30] that are not available in large-scale genetic analysis software.

Here, we considered farm and monthly mean temperature as environmental variables and present a comprehensive study of G × E for weaning weight (WW), yearling weight (YW), 18-month body weight (18 BW), and 24-month body weight (24 BW) in more than 1300 Huaxi cattle. While using LD score regression methods ensured that the introduction of the robust estimator could appropriately control for the aforementioned inflation and confounding of GWEISs in all experimental scenarios, we first explored SNP–environment interactions between all environmental variables and genome-wide 770 K chip SNPs. We then sought to elucidate the relevant biological mechanisms that may control G × E by testing whether individual SNP–environment interaction effects are overrepresented in specific genes or gene sets. In parallel with all interaction-based analyses, we performed traditional GWASs for the four growth traits in the same population to assess the agreement between the top interaction effects and the corresponding main effects. Our results revealed novel interactions between genetic loci, genes, and candidate biological mechanisms associated with growth traits, providing new insights into environmental adaptation and genetic mechanism resolution of growth traits in beef cattle. 

## 2. Materials and Methods

### 2.1. Animal Resource and Phenotypes Recording

A total of 1350 individuals in this study were derived from the Chinese Simmental beef cattle core populations born between 2009 and 2019 in China. All the cattle lived on seven different farms located throughout China, where they were fed a similar diet consisting of silage, maize, beer grain, and soybean dregs. Phenotypic records of growth traits, namely the weaning weight (WW), the yearling weight (YW), the 18-month body weight (18 BW), and the 24-month body weight (24 BW), were examined. All the growth traits in the study were measured within one month before or after the growth time point, using uniform measurement specifications in the beef cattle performance test.

### 2.2. Genotyping and Quality Control

Blood samples for the experimental population were collected during the periodic quarantine inspection of the farms. Genomic DNA was isolated from blood samples using the TIANamp Blood DNA Kit (Tiangen Biotech Co., Ltd., Beijing, China), and the high-quality DNAs with the A260/280 ratio with a range of 1.8–2.0 were considered for further analysis. In this study, the Illumina BovineHD Beadchip with 774,660 SNPs (Illumina Inc., San Diego, CA, USA) was used for qualified DNAs genotyping and Illumina’s Infinium II Assay was selected as the genotyping platform. The SNPs were uniformly distributed on the whole bovine genome with a mean inter-marker space of 3.43 kb. SNP chips were scanned and analyzed using the Infinium GenomeStudio software v2.0 (Illumina Inc., San Diego, CA, USA). PLINK v1.9 (http://zzz.bwh.harvard.edu/plink/, accessed on 1 July 2021) was used for quality control of SNPs according to the following empirical excluded criteria: (1) minor allele frequency (MAF) < 0.05; (2) SNP call rate (CR) < 90%; (3) Hardy–Weinberg equilibrium value *p* < 1 × 10^−6^; (4) Mendelian error of SNP genotype above 2%; (5) individuals with more than 10% SNPs deletion; (6) SNP marker sites with missing chromosomal location information. All the misplaced and duplicated SNPs were also excluded from the analysis. Ultimately, 598,430 SNPs with an average marker interval of 3 kb on 29 autosomal chromosomes remained for subsequent analysis.

### 2.3. Environmental Factors

We considered farms and the average monthly temperature during the growth period of each individual as environmental factors. As for the farm factor, it can be regarded as a limited-dimensional discrete variable, all cattle were sourced from seven different farms in various regions of China, including Changchun Xinmu (CCXM, longitude 125.464848° E and latitude 43.71698° N), Jilin Dexin (JLDX, longitude 122.946° E and latitude 45.565° N), Tongliao Keerqin (KEQ, longitude 122.040° E and latitude 43.860° N), Aokesi farm in Inner Mongolia (AKS, longitude 119.316° E and latitude 46.278° N), Henan Dingyuan (HNDY, longitude 114.022° E and latitude 34.894° N), Shayang Hanjiang (SYHJ, longitude 112.584° E and latitude 30.864° N), and Yunnan Zhongchu (YNZC, longitude 102.986° E and latitude 25.180° N). The monthly average temperature was collected at the nearest climatological station (National Oceanic and Atmospheric Administration, NOAA, Washington, DC, USA, https://www.noaa.gov (accessed on 14 October 2022)) to each farm and was calculated by averaging the monthly temperatures of each individual from birth to phenotype recording, which can be considered as a continuous variable since the monthly mean temperature of each individual is unique and calculated independently.

### 2.4. GWEIS

A linear interaction model in R (v4.1.3) was used to analyze SNP–environment interactions. The heteroscedasticity of the residuals makes genotype-by-environment interaction association studies (GWEIS) test statistics particularly vulnerable to erroneous inflating of test statistics. We used the sandwich estimator, also known as Huber–White estimated standard errors [30], to handle this. The sandwich estimator permits a distinct residual variance term across data, which is estimated using the squared residuals, in contrast to model-based standard errors, which are computed using a single residual variance term for all observations.

Our script is an adaptation of a PLINK R plugin originally developed by Almli et al. [31], which performs a joint test of SNP and SNP–environment interaction effects (https://epstein-software.github.io/robust-joint-interaction (accessed on 22 August 2022)). Beyond run-time optimization, we computed *p*-values for the gene-environment interaction (rather than the joint test of SNP main and interaction effects, as performed initially), and included covariate–SNP and covariate–environment interactions in addition to covariate main effects. Autosomal SNPs were coded as 0, 1, and 2, representing the homozygous minor, heterozygous, or homozygous major genotypes, respectively. We thus implemented the following linear regression model for every SNP:

(1) $$y_{i}=\mu_{0}+\beta_{g}G_{i}+\beta_{e}E_{i}+\beta_{g\times e}G_{i}E_{i}+\beta_{c}{C^{\prime }}_{i}+\beta_{c\times g}{C^{\prime }}_{i}G_{i}+\beta_{c\times e}{C^{\prime }}_{i}E_{i}+\varepsilon_{i}$$

where $y_{i}$ represents the phenotype measure for any individual *i*, $G_{i}$ the SNP allele count, and $E_{i}$ the environmental measure. $C_{i}$ is a *k*  *×*  *n* vector of covariates, with *k* equalling the total number of covariates; $\varepsilon_{i}$ the residual; and ′ denotes the transpose. The intercept ($\mu_{0}$) and betas for the SNP ($\beta_{g}$), environment ($\beta_{e}$), and SNP–environment interaction term ($\beta_{g\times e}$) are all scalars, while the betas for the covariate–environment ($\beta_{c\times e}$) and covariate–SNP ($\beta_{c\times g}$) interactions are *k*  *×*  *1* vectors. The parameter of interest here is $\beta_{g\times e}$: the beta for the SNP–environment interaction.

We included age, sex, birth weight, and 20 PCs (Principal Component) as covariates. As recommendations or standards regarding the number of PCs that should be included typically pertain to analyses of main effects, we could not rule out the possibility that potentially more complex confounding effects of ancestry could emerge when analyzing interactions; therefore, we opted for a more cautious approach by including up to 20 covariates.

### 2.5. GWAS

Univariate linear mixed model analyses were performed for the WW, YW, 12 BW, or 24 BW in GEMMA v0.98.5 software using the same set of covariates as in the GWEIS:

(2) y=1μ+Xβ+Zu+ε

where y is an n-vector of phenotypes; μ is the overall mean; X is the incidence matrix of genotypes; β represents genotype effects; Z is the random effects design matrix; u is an n-vector of random additive genetic effects; and ε is an n-vector of random residual. Assuming that $u\sim N(0,G\sigma_{a}^{2})$ and $e\sim N(0,I\sigma_{e}^{2})$, G is the n × n genomic relationship matrix; $\sigma_{a}^{2}$ is the additive genetic variance; $\sigma_{e}^{2}$ is the residual variance component; I is an n × n identity matrix. 

### 2.6. Gene-Based and Gene-Set Analyses

To investigate whether SNP–environment interaction signals tend to cluster within gene regions and whether G × E-containing genes are enriched in specific biological pathways, we performed genome-wide gene-based analysis as well as gene-set analysis using MAGMA (v1.10) [32], using the initial GWEIS *p*-values as input to obtain *p*-values of genes for gene-set analysis. To allow the inclusion of nearby potential regulatory SNPs, we used windows of 50 kb upstream and downstream [33] of the transcription start and stop sites, respectively. The positions of 23,431 genes were obtained from Ensembl (Bos taurus ARS-UCD1.2 (ftp://ftp.ensembl.org/pub/release-101/fasta/bos_taurus/DNA/ (accessed on 15 November 2022)), of which 23,305 genes mapped at least one SNP. While a total of 1342 definitions of gene sets related to cattle were obtained from Reactome (https://reactome.org/ (accessed on 26 November 2022)), 1283 of them reached the condition of containing multiple genes for analysis. For both analyses, we used the default settings of MAGMA, with gene-based and gene-set analyses using the ‘SNPwise-mean’ model and a competitive framework, respectively.

## 3. Results

### 3.1. GWEIS Test Statistics

**Figure 1:**
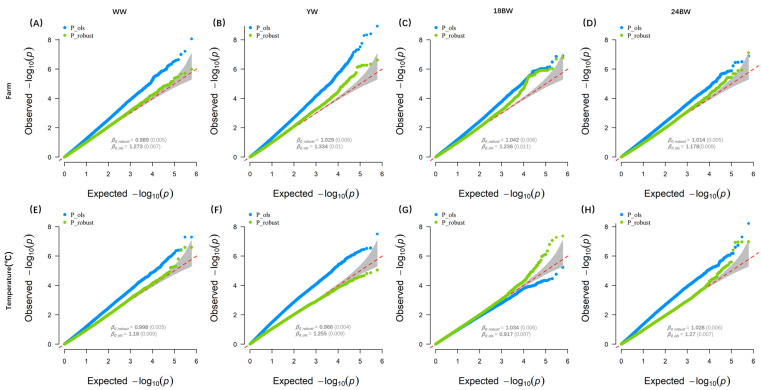
QQ-Plots for the GWEIS analysis using traditional model-based (blue) and robust (green) variance estimator. (**A**–**D**) QQ-Plots of −log10 (*p*-value) for the weaning weight (WW), the yearling weight (YW), the body weight of 18 months (18 BW), and the body weight of 24 months (24 BW) across farms, respectively. (**E**–**H**) QQ-Plots of −log10 (*p*-value) for WW, YW, 18 BW, and 24 BW across temperatures, respectively. *β*_0_ denotes LD score intercepts (with standard errors) from LDSC.

In order to solve the mentioned risk of spurious inflation of the GWEIS test statistic previously [26,31], we examined SNP–environment interactions in a linear regression model using the R script, computing t-statistics for the interaction coefficients using robust standard errors in the form of the Huber–White sandwich estimator, and we included SNP–covariate and environment–covariate interaction effects in the model to account for any potential confounding covariate interactions. We compared this method with the traditional model test statistic by obtaining LD score regression intercepts [34] and plotting the observed −log10 *p*-values against the expected null distribution in the form of QQ-plots for results. When relying on the traditional model-based standard errors, we could observe the presence of mild inflation in most of the scenarios. The intercepts of the LD score for the traditional model-based results ranged from 0.917 to 1.334, and the QQ-plots indicated deviations of the *p*-values of the weakly correlated SNP from the expected distribution in most cases. However, this is not the case for the robust results, where the QQ-plots show no signs of inflation and the LD score intercepts were all below 1.042 (Figure 1).

### 3.2. G × E Interacting SNPs Detected by GWEIS

We analyzed the single SNP-by-environment interactions between each of WW, YW, 18 BW, and 24 BW (N  = 777–1187; Table 1) and a total of 598,430 SNPs (minor allele frequency > 0.05; call rate > 0.9; see Section 2), from which we identified 29 independent SNPs (r^2^  <  0.8) for the four growth traits that showed G × E interaction effects at the standard genome-wide significance threshold (*p*  <  1/598,430 = 1.67 × 10^−6^; Table 2 and Figures S1–S8). As a result, a total of 12 and 10 candidate interaction genes from farms and monthly average temperature were located by those significant SNPs, respectively, and 4, 2, 10, and 7 of them were associated with WW, YW, 12 BW, and 24 BW (Table 2). Of these, with further Bonferroni correction (*p* < 0.05/598,430 = 8.36 × 10^−8^), seven SNPs were identified in the two environments, and the four traits remained significant and enriched to four genes: Proprotein Convertase Subtilisin/Kexin Type 5 (*PCSK5*), Filamin B (*FLNB*), Sterile Alpha and TIR Motif Containing 1 (*SARM1*), and Solute Carrier Family 46 Member 1 (*SLC46A1*). 

**Figure 2:**
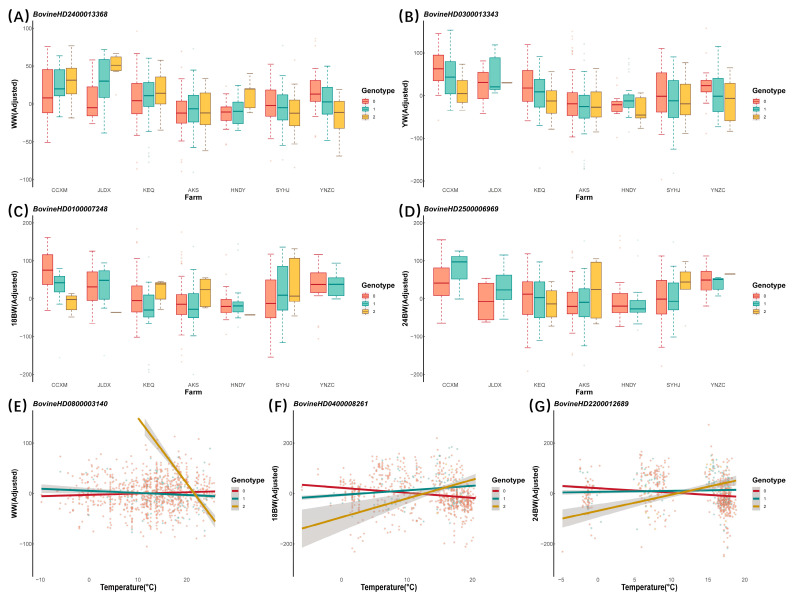
Interaction plots of phenotypes changing over environment factors for different genotypes of significant SNPs. The 0, 1, and 2 corresponding to homozygous reference, heterozygous, and homozygous alternative genotypes are drawn with red, turquoise, and golden. The *y*-axis is the phenotype of growth traits that have been adjusted for sex, first birth weight, and age of days. The *x*-axis represents environmental variables, where the environmental variables are farms in the (**A**–**D**) and temperature in the (**E**–**G**). Only a single significant SNP is shown for each trait-environment case, while other significant SNPs are shown in Supplemental Figures.

Furthermore, we further visualized genotype re-ranking by plotting the phenotypic performance in response to environmental changes in the 29 identified independent SNPs in Figure 2 and Figures S9–S15. The 0, 1, and 2 corresponding to homozygous reference, heterozygous, and homozygous alternative genotypes were drawn with red, dark green, and golden. In this case, the superior genotypes of those SNPs would rearrange with either discrete (farm, Figure 2A–D and Figures S9–S11) or continuous (temperature, Figure 2E–G and Figures S12–S15) environmental variables. For instance, BovineHD2400013368 genotypes are ranked 2 > 1 > 0 in some farms (high latitudes such as CCXM or JLDX) and 0 > 1 > 2 in others (low latitudes such as SYHJ or YNZC) for the WW trait. The genotypes of BovineHD2400013368 exhibited negative pleiotropy and had an opposite effect across temperatures (as manifested in slope differences) for the WW trait. This verified the existence of G × E interaction regarding the change in traits-farm and/or traits-temperature.

These results contrast with traditional GWAS using the same covariates as GWEISs for the four growth traits, where eight independent significant SNPs were detected. For the 29 SNPs that showed significant G × E interactions at the standard genome-wide significance threshold (*p* < 1.67 × 10^−6^), we did not find any indication of significant main effects in GWAS (*p* > 0.05; Table 2).

### 3.3. Genes and Gene Sets Implicated by SNP–Environment Interactions

**Figure 3:**
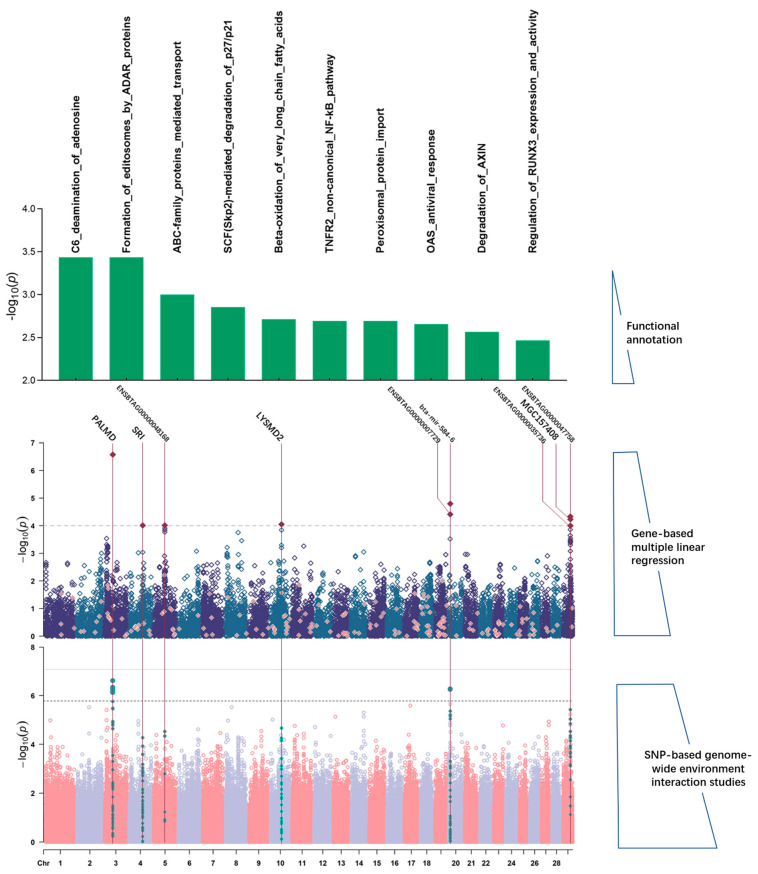
Overview and results of SNP-based, gene-based, and pathway enrichment G × E interaction studies for YW-Farm. **Bottom**: Manhattan plot showing the −log10 *p*-values of SNP–environment interaction from the GWEIS. Gene labels have been annotated to suggestively significant (*p* < 1/598,430 = 1.67 × 10^−6^) intragenic SNPs with the lowest *p*-value. Manhattan plot showing each genomic risk locus which maps to the significant interacting genes in gene-based analysis (green). **Middle**: Manhattan plot showing the −log10 *p*-values for the MAGMA gene analysis (based on the GWEIS SNP–environment interaction results). Suggestively significant genes (*p* < 1 × 10^−4^) have been annotated with claret. The pink dots represent the genes contained in the gene set used for subsequent mapping. **Top**: Pathways in the top 10 of −log10 *p*-value in MAGMA gene-set analysis enrichment.

To facilitate functional interpretation of the GWEIS results, we sought to clarify whether genome-wide SNP–environment interaction effects tended to aggregate within specific genes and gene sets (Figure 3). We performed genome-wide gene-based association studies for 23,305 genes conducted in Multi-Marker Analysis of GenoMic Annotation (MAGMA) using the interaction *p*-values from all the eight GWEISs as input (Figure 3 and Figures S16–S23). As a result, we found a total of 20 genes that reached standard genome-wide significance (*p*  <  1 × 10^−4^; Table 3); only one gene (Palmdelphin (*PLAMD*) in Farm-YW) remained significant under the further Bonferroni correction (*p*  <  0.05/23,305 = 2.14 × 10^−6^). In addition, three significant genes (*SMAD2* (SMAD Family Member 2), *PALMD*, *MECOM* (MDS1 and EVI1 Complex Locus) in Farm-WW, Farm-YW, and Farm-18 BW, respectively) had been identified in the previous GWEIS analysis. Similar to the SNP-level results, the concordance between suggestive main and interaction effects at the gene level was low, and no genes were detected to achieve suggestibility significance in the main effect genes for all scenarios.

Further, to determine whether the most strongly associated genes for any environment (including sub-significant genes) tended to be overrepresented within particular pathways, we performed competitive gene-set and gene-property analyses in MAGMA using the results from the GWEIS-based gene-wise analyses as input. These analyses concerned 1283 gene sets (Reactome; see Section 2). At a *p*-value threshold of 1 × 10^−4^, 7 pathways from five scenarios were significant (Table 4). All of these pathways remain significant under the additional Bonferroni correction (*p*  <  0.05/1347 = 3.71 × 10^−5^; Table 4).

## 4. Discussion

In this study, we explored genome-wide gene–environment interactions of four growth traits (including weaning weight WW, yearling weight YW, the weight of 18 months 18 BW, and the weight of 24 months 24 BW) across two environmental factors (farm and mean monthly temperature). From all SNP-, gene-, and gene-set-based analyses, we detected independent 29 SNPs, 20 genes, and 7 gene sets that showed G × E effects at standard genome-wide significance thresholds, of which 20 SNPs, 5 genes, and 7 gene sets, respectively, remained significant under the further Bonferroni correction.

An important issue in the interaction model is heteroskedasticity, which refers to the observation that the variance of trait outcome differs among levels of environmental exposure, and this phenomenon violates the assumption that trait variance should be the same across individuals (homoskedasticity) in linear regression, with the result that substantial genome-wide inflation occurs [31]. Here, we incorporated robust standard errors often referred to as Huber–White, sandwich, or heteroskedasticity consistency errors [35,36] in model analysis, which are often considered to deal with misspecification of mean and variance assumptions in statistical models due to its heteroskedasticity insensitivity. Robust SEs are widely used in econometric, anthropomorphic [37], and biomarker [38] studies for correcting inflation, and also demonstrated effectiveness in gene–environment interaction studies [27,28,39]. The LD score regression results showed that the robust test corrected deviations present in model-based tests regardless of whether the environmental factors were discrete or continuous variables (manifested in this study as farm and temperature), demonstrating the strategy’s effectiveness and feasibility of GWIES in animal G × E analysis.

In contrast to previous studies, which suggested that the power for main effect detection was higher than that for interaction effects [40,41,42], we detected more significant interactions using GWEIS than those detected in a traditional GWAS on growth traits, which only detects eight independent SNPs with the same significance threshold, implying that the detection of traditional main effects compared to interaction effects may be more sensitive to the size of the sample population and individual source consistency. That is, when experimental samples come from uneven environments, the main effect of genotype may be obscured by the G × E that occurs due to environmental diversity. In addition, the interacting SNPs we identified under the standard significance threshold of GWEIS showed no evidence of suggestive main effects in GWAS, nor in the subsequent gene-based and gene-set analyses, implying that important interacting effects might be overlooked in the detection of main effects.

The farm effect is complex and should be emphasized in animal production since it involves many potential environmental factors that affect animal growth and development, such as management, nutrition level, and climate. Under all the GEWIS studies on growth traits with the farm as the environmental factor, we localized a total of 12 coding genes, and 3 of them showed significant interaction effects in the next gene-based analyses, namely *SMAD2*, *PALMD*, and *MECOM*, which were distributed on BTA 24, BTA 3, and BTA 1 associated with WW, 12 BW, and 18 BW, respectively. *SMAD2* regulates a variety of cellular processes by mediating transforming growth factor (TGF)-β signaling and is involved in the regulation of reproduction and embryonic development in cattle [43]. Meanwhile, *SMAD2* can regulate the level of myogenic inhibitors in the biological organism, thus participating in myofiber proliferation and affecting growth and development [44,45]. Furthermore, in a study of *SAMD2* expression in thoracic aortic aneurysms (TAAs) of different etiologies, *SAMD2* was found to be epigenetically regulated, implying the presence of environmental interactions [46]. The palmdelphin (*PALMD*) gene was differentially expressed between high- and low-yielding Holsten cattle and thus considered to be associated with milk production traits in cattle [47], while a study on production traits in pigs [48] found that gene polymorphisms of *PALMD* had a significant effect on backfat thickness. The *PALMD* also promotes basal progenitors (BPs) proliferation via integrin signaling, which is a necessary pathway for the evolutionary expansion of the mammalian neocortex [49]. The *MECOM* gene, also named *EVI1*, encodes a protein that is an oncoprotein that may be involved in hematopoiesis, apoptosis, development, and cell differentiation and proliferation [50], and has been identified as a candidate gene associated with lung weight and kidney weight in cattle in a previous GWAS on bovine visceral organ weights by An et al. [51]. In addition, *MECOM* has also been reported as a candidate gene associated with lung function and longissimus dorsi development in cattle [52,53]. Similarly, in a human blood-pressure-related gene–environment interaction study, *MECOM* exhibited a strong interaction with smoking [54]. These functional studies suggest that these three genes may be important target genes that play important roles in the regulation of growth and development in beef cattle and receive gene–environment interactions. 

Contrary to expectations, our temperature-related G × E studies did not find overlapping genes between SNP- and gene-based interaction analyses, unlike the results from farm-related studies. Despite this lack of overlap, the 11 and 5 genes identified in SNP- and gene-based interaction analyses under temperature G × E conditions were predominantly associated with crucial aspects of growth and development, such as fat deposition (*HMBOX1* [55]; *FLNB* [56]), feed efficiency (*BNIP1* [4]), and heat stress (*SARM1* [56]). A possible explanation for this phenomenon is that, compared to the single unidimensional variable of temperature, the farm variable not only encompasses climatic factors but also considers environmental effects that are difficult to measure, such as feed quality and management, and this integrative environmental factor guarantees the ability to detect environmentally sensitive loci. Another explanation is that the temperature was set as a continuous variable in this study; however, the animals themselves develop adaptations that determine that they are insensitive to temperature fluctuations within a certain interval, so the effect of G × E may be more pronounced in the early developmental stages [57]. These findings support the importance of including environmental interaction effects in multi-population genomic prediction, and using the G × E SNPs detected in this study to support breeding decisions may yield benefits for animal production performance.

Gene-set analysis was considered to identify biological processes that were significantly regulated by the collective influence of subtle perturbations to multiple functional genes [58]. In our study, gene-set-based pathway analyses using MAGMA yielded seven significant gene sets and remained significant with additional Bonferroni correction threshold (*p* < 0.05/1283 = 3.90 × 10^−5^), clustered around functional pathways related to growth and development such as cell disaggregated proliferation, fatty acid β-oxidation, gap junctions, and keratin sulfate. For instance, polo-like kinases (Plks) are cell cycle-regulating serine/threonine kinases that play important roles in cell–cell mitosis, spindle formation, and cytoplasmic division [59]. Gap junctions are specialized intercellular junctions between multiple animal cell types that affect the development of embryos, tissues, and organs [60,61]. Gene-set analysis of GWEIS data considers the G × E interaction contained in most genetic makers to identify functionally annotated collections of genes enriched for phenotypic association, and although the results may be variably driven by the diversity of gene sets collected, it is an attractive strategy for gaining insight into biological mechanisms.

## 5. Conclusions

In this study, we introduced a robust estimator into our model to control inflation caused by heteroskedasticity and performed genome-wide gene–environment interactions association analysis for four growth traits and two environmental factors in beef cattle. Through this approach, we detected a few genetic markers affecting growth traits that did not show significant main effects in traditional GWAS, demonstrating that functional loci may have non-additive effects across genotypes but are obliterated by environmental means. Further follow-up testing on whether these reciprocal loci tend to cluster in genes or gene sets reveals the biological mechanisms by which genetic factors modulate the response to environmental influences on performance. Our results revealed novel interactions between genetic loci, genes, and candidate biological mechanisms associated with growth traits, providing new insights into environmental adaptation and the resolution of genetic mechanisms influencing growth traits in beef cattle. These findings prompt further investigation into how these genetic loci can be effectively integrated into breeding programs to enhance resilience and performance in diverse environmental conditions.

## Figures and Tables

**Table 1:** Descriptive statistics (Mean ± SD, kg) on growth traits of Huaxi Cattle.

Farms	N	WW		YW		18 BW		24 BW	
		Male	Female	Male	Female	Male	Female	Male	Female
CCXM	70	258.75 ± 51.38	279.31 ± 36.58	478.04 ± 48.73	391.46 ± 35.55	684.5 ± 83.42	494.31 ± 47.76	839.96 ± 82.45	589.08 ± 68.9
JLDX	35	228.74 ± 32.81		470.53 ± 61.07		637.91 ± 68.14		807.55 ± 68.89	
KRQ	238	234.54 ± 61.26	213.48 ± 30.05	444.54 ± 55.11	357.13 ± 38.95	645.33 ± 94.81	459.31 ± 36.73	817.68 ± 82.2	522 ± 38.38
AKS	379	165.2 ± 64.1	152.83 ± 49.64	366.12 ± 69.66	274.5 ± 57.64	594.59 ± 102.3	382.82 ± 46.78	769.97 ± 155.6	447.67 ± 52.59
HNDY	72	184.26 ± 27.37	171.93 ± 34.29	495.78 ± 43.5	475.83 ± 49.14	711.5 ± 47.91	534.86 ± 47.8	880.74 ± 45.06	574.5 ± 58.82
SYHJ	437	194.97 ± 24.12	208.99 ± 22.86	369 ± 39.58	285.03 ± 30.32	559.67 ± 76.88	372.94 ± 35.19	740.91 ± 68.86	457.19 ± 46.13
YN	119	243.12 ± 56.2	232.91 ± 41.74	458.17 ± 47.55	398.09 ± 30.34	685.6 ± 30.9	506.43 ± 50.12	814.43 ± 54.47	588.17 ± 58.02
Total	1350	1187		959		836		777	

Name of traits: WW weaning weight, YW yearling weight, 18 BW body weight of 18 months, and 24 BW body weight of 24 months.

**Table 2:** Significant SNPs with G × E interactions identified in the GWEIS.

Environment	Trait	SNP	BTA ^1^	POS ^2^	Gene	P_GWEIS_	P_GWAS_
Farms	WW	BovineHD0300020017	3	67,574,825	*AK5*	1.03 × 10^−6^	0.614
		BovineHD2400013368	24	47,983,708	*SMAD2*	1.11 × 10^−6^	0.243
	YW	BovineHD0300013343	3	43,785,038	*PALMD*	2.45 × 10^−7^	0.975
		BovineHD2000001962	20	6,217,212	*MIR584-6*	5.39 × 10^−7^	0.407
	18 BW	BovineHD2400007024	24	25,954,580	*DSG2*, *DSG3*	1.71 × 10^−7^	0.614
		BovineHD0900021567	9	77,291,417	*NHSL1*	2.06 × 10^−7^	0.853
		ARS-BFGL-NGS-58606	21	34,730,593	*CYP11A1*, *CCDC33*	9.11 × 10^−7^	0.271
		BTB-01643687	4	8,200,949	*CDK14*	9.50 × 10^−7^	0.667
		BovineHD0100028293	1	99,131,999	*MECOM*	1.23 × 10^−6^	0.011
		BovineHD1600021531	16	75,288,520	*-*	1.42 × 10^−6^	0.600
		BovineHD0100007248	1	24,519,328	*ROBO2*	1.59 × 10^−6^	0.249
	24 BW	BovineHD2500006969	25	24,706,966	*-*	7.55 × 10^−8^ *	0.982
		BovineHD0200033391	2	115,898,667	*RHBDD1*	1.20 × 10^−6^	0.412
Temperature	WW	BovineHD0700013465	7	46,699,643	*-*	2.50 × 10^−7^	0.304
		BovineHD0800003140	8	9,686,754	*HMBOX1*	4.14 × 10^−7^	0.165
		BovineHD1900009199	19	31,126,798	*DNAH9*	1.57 × 10^−6^	0.277
	18 BW	BovineHD0400011664	4	42,477,921	*-*	4.30 × 10^−8^ *	0.368
		BovineHD0800015785	8	52,604,454	*PCSK5*	5.20 × 10^−8^ *	0.796
		BovineHD2000001524	20	4,793,312	*BNIP1*	8.65 × 10^−8^	0.712
		BovineHD0600020848	6	74,944,109	*-*	1.61 × 10^−7^	0.328
		BovineHD0900023319	9	83,628,109	*-*	7.59 × 10^−7^	0.857
		ARS-BFGL-NGS-96591	10	4,157,289	*-*	8.63 × 10^−7^	0.484
		BovineHD0800014102	8	47,106,702	*TRPM3*	9.57 × 10^−7^	0.761
		BovineHD0400008261	4	28,773,445	*-*	1.07 × 10^−6^	0.554
	24 BW	BovineHD2200012689	22	43,774,319	*FLNB*	6.15 × 10^−8^ *	0.021
		BovineHD2900006104	29	21,265,657	*-*	6.42 × 10^−8^ *	0.985
		BovineHD2000005848	20	19,541,201	*MIR582*	6.61 × 10^−8^ *	0.641
		BovineHD1900005841	19	20,432,774	*SARM1*, *SLC46A1*	7.05 × 10^−8^ *	0.581
		BovineHD1000001235	10	4,037,591	*PGGT1B*, *CCDC112*	2.46 × 10^−7^	0.729

SNPs from all GWEIS analyses with a *p*-value below the standard genome-wide significance threshold (*p* < 1/598,430 = 1.67 × 10^−6^). Name of traits: WW weaning weight, YW yearling weight, 18 BW body weight of 18 months, and 24 BW body weight of 24 months. ^1^ *Bos Taurus* Autosome. ^2^ POS: Position (bp) on ARS-UCD1.2. * = remain significant under the Bonferroni correction (*p* < 0.05/598,430 = 8.36 × 10^−8^).

**Table 3:** Genes implicated in MAGMA gene analysis.

Environment	Trait	Gene	BTA	POS_START_	POS_STOP_	P_GWEIS_	P_GWAS_
Farms	WW	*SMAD2*	24	47,921,047	48,072,060	4.58 × 10^−6^	0.466
		*IQCN*	7	4,825,678	4,954,688	1.57 × 10^−5^	0.500
		*CIST1*	7	4,793,442	4,960,673	5.25 × 10^−5^	0.579
	YW	*PALMD*	3	43,638,103	43,798,131	2.66 × 10^−7^ *	0.773
		*MIR584-6*	20	6,189,918	6,289,993	1.59 × 10^−5^	0.231
		ENSBTAG00000007729	20	6,155,300	6,256,217	3.88 × 10^−5^	0.295
		ENSBTAG00000047758	29	39,060,430	39,165,394	4.64 × 10^−5^	0.005
		*MGC157408*	29	39,407,513	39,516,695	5.89 × 10^−5^	0.024
		*LYSMD2*	10	58,681,531	58,794,506	8.85 × 10^−5^	0.264
		ENSBTAG00000048168	5	59,242,315	59,343,254	9.68 × 10^−5^	0.302
		*SRI*	4	72,724,525	72,846,824	9.72 × 10^−5^	0.059
		ENSBTAG00000035736	29	39,491,478	39,600,943	9.98 × 10^−5^	0.008
	18 BW	*MECOM*	1	99,040,972	99,193,289	1.57 × 10^−5^	0.016
		*KREMEN2*	25	2,322,471	2,427,363	3.49 × 10^−5^	0.999
	24 BW	ENSBTAG00000046633	21	69,135,855	69,236,382	6.31 × 10^−5^	0.669
Temperature	WW	*ABCC10*	23	16,911,898	17,031,880	2.08 × 10^−5^	0.029
		*DLK2*	23	16,931,806	17,036,060	2.10 × 10^−5^	0.029
		*TJAP1*	23	16,951,348	17,075,374	4.34 × 10^−5^	0.109
		*LRRC73*	23	16,975,858	17,078,655	6.35 × 10^−5^	0.120
		*YIPF3*	23	16,981,093	17,085,360	8.49 × 10^−5^	0.127

Results from MAGMA gene analysis of 23,305 genes, using the GWEIS interaction *p*-values as input (*p* < 1 × 10^−4^); * = gene remain significant under the additional Bonferroni correction for the number of environments analyzed (*p* < 0.05/23,305 = 2.14 × 10^−6^).

**Table 4:** Gene sets enriched in the MAGMA gene-set analyses.

Environment	Trait	Pathway	P_GWEIS_	P_GWAS_
Farms	WW	Mitotic Telophase/Cytokinesis	7.66 × 10^−10^ *	0.106
		Polo-like kinase-mediated events	1.27 × 10^−5^ *	0.162
	24 BW	Mitochondrial Fatty Acid Beta-Oxidation	5.16 × 10^−7^ *	0.288
Temperature	WW	Reduction in cytosolic Ca++ levels	2.29 × 10^−5^ *	0.906
	YW	Formation of annular gap junctions	9.08 × 10^−6^ *	0.230
		Gap junction degradation	1.87 × 10^−5^ *	0.429
	24 BW	Keratan sulfate degradation	1.59 × 10^−8^ *	0.069

Results from MAGMA analysis of 1283 Reactome gene sets (*p* < 1 × 10^−4^). * = gene sets that remain significant under the Bonferroni correction (*p* < 0.05/1283 = 3.90 × 10^−5^).

## Data Availability

The datasets are available upon request from the National Center of Beef Cattle Genetic Evaluation, Beijing, China (Email: pingguzhongxin@126.com).

## Supplementary Materials

The following supporting information can be downloaded at: https://www.mdpi.com/article/10.3390/ani14111695/s1, Figure S1: Manhattan plot of GWEIS results for Farm-WW; Figure S2: Manhattan plot of GWEIS results for Farm-YW; Figure S3: Manhattan plot of GWEIS results for Farm-18BW; Figure S4: Manhattan plot of GWEIS results for Farm-24BW; Figure S5: Manhattan plot of GWEIS results for Temperature-WW; Figure S6: Manhattan plot of GWEIS results for Temperature-YW; Figure S7: Manhattan plot of GWEIS results for Temperature-18 BW; Figure S8: Manhattan plot of GWEIS results for Temperature-24 BW; Figure S9: Interaction plot of significant SNPs for Farm-WW; Figure S10: Interaction plot of significant SNPs for Farm-YW; Figure S11: Interaction plot of significant SNPs for Farm-18 BW; Figure S12: Interaction plot of significant SNPs for Farm-24 BW; Figure S13: Interaction plot of significant SNPs for Temperature-WW; Figure S14: Interaction plot of significant SNPs for Temperature-18 BW; Figure S15: Interaction plot of significant SNPs for Temperature-24 BW; Figure S16: Manhattan plot of MAGMA gene analysis results for Farm-WW; Figure S17: Manhattan plot of MAGMA gene analysis results for Farm-YW; Figure S18: Manhattan plot of MAGMA gene analysis results for Farm-18 BW; Figure S19: Manhattan plot of MAGMA gene analysis results for Farm-24 BW; Figure S20: Manhattan plot of MAGMA gene analysis results for temperature-WW; Figure S21: Manhattan plot of MAGMA gene analysis results for temperature-YW; Figure S22: Manhattan plot of MAGMA gene analysis results for temperature-18 BW; Figure S23: Manhattan plot of MAGMA gene analysis results for temperature-24 BW.
