# Supplementary material for: Genome-Wide Gene–Environment Interaction Analysis Identifies Novel Candidate Variants for Growth Traits in Beef Cattle
Source: Animals (Basel). 2024 Jun 5;14(11):1695. doi: 10.3390/ani14111695 (PMC11171348; doi:10.3390/ani14111695)
Supplement: Supplementary file 1 [file animals-14-01695-s001.zip › animals-2991413-supplementary.pdf]

Supplementary Figures

This section contains relevant analysis plots organized by growth traits and environments. For all environments, there are Manhattan plots for the GWEIS and gene-analysis, and for those with any genome-wide significant SNPs form the GWEIS, there are also annotated interaction plots.

GWEIS Manhattan plot

Shows the -log10 p-values for the SNP interaction with the environment in question. Gene names have been annotated to suggestively significant (p <1/598,430 = 1.67e-6) independent SNPs with the lowest p-value.

Interaction plots

These plots show the adjusted growth trait phenotypes at any given combination of alleles and environmental factor.

Gene analysis Manhattan plot

Shows the -log10 p-values for the MAGMA gene analysis (based on the GWEIS SNP-environment interaction results). Suggestively significant genes (p < 1e-4) have been annotated.

GWEIS Manhattan plot

Shows the -log10 p-values for the SNP interaction with the environment in question. Gene names have been annotated to suggestively significant (p <1/598,430 = 1.67e-6) independent SNPs with the lowest p-value.

Farm-Weaning weight (WW)

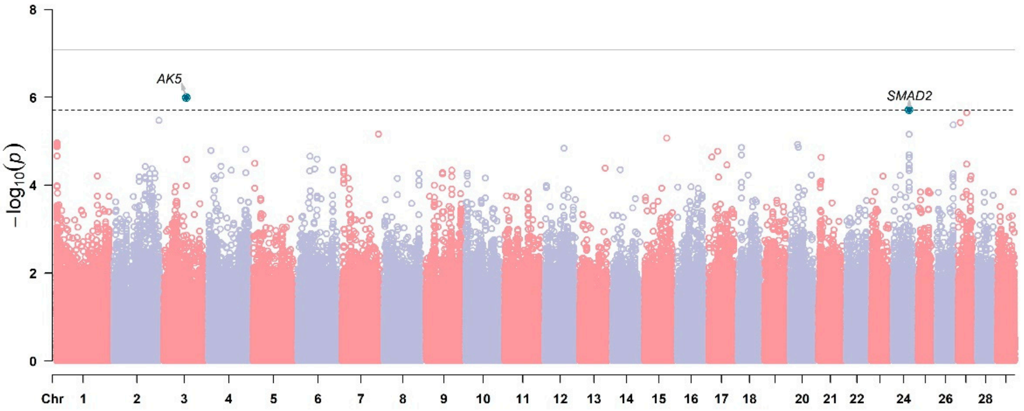

Supplementary Figure S1. Manhattan plot of GWEIS results for Farm-WW. Manhattan plot showing the -log10 SNP-environment interaction p-values from the GWEIS. Gene labels have been annotated to suggestively significant (p < p <1/598,430 = 1.67e-6) independent SNPs with the lowest p-value.

Farm-Yearling weight (YW)

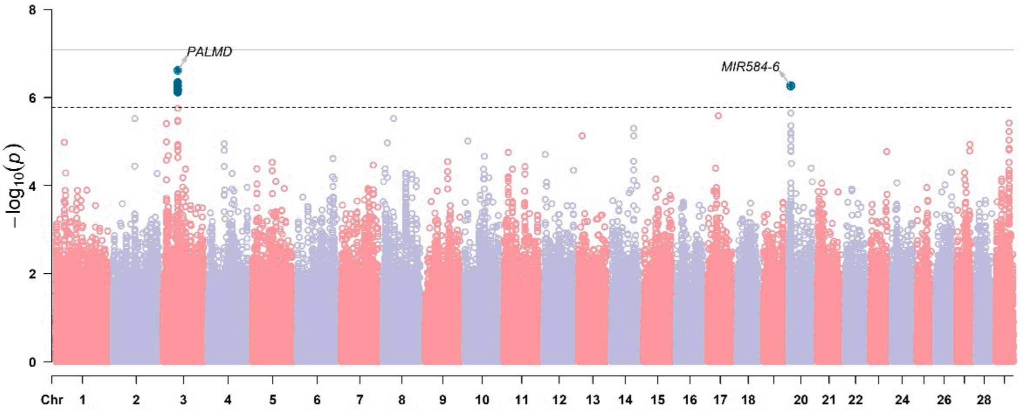

Supplementary Figure S2. Manhattan plot of GWEIS results for Farm-YW. Manhattan plot showing the -log10 SNP-environment interaction p-values from the GWEIS. Gene labels have been annotated to suggestively significant (p < p <1/598,430 = 1.67e-6) independent SNPs with the lowest p-value.

Farm-Weight of 18 months (18BW)

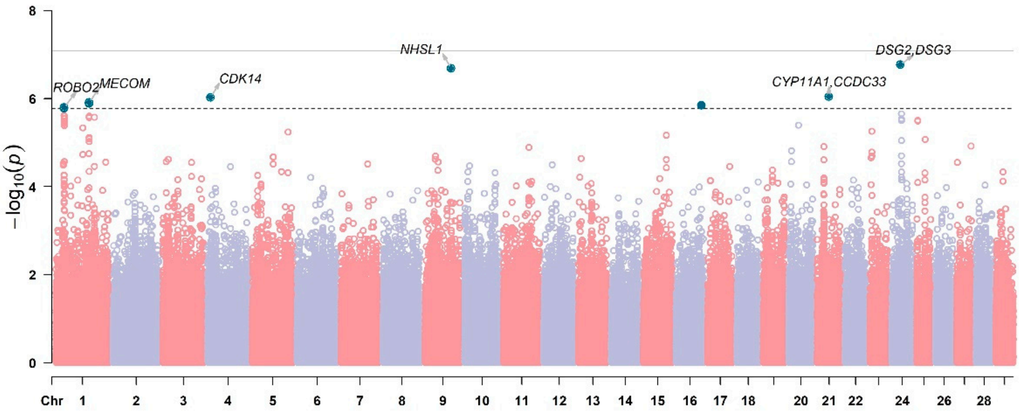

Supplementary Figure S3. Manhattan plot of GWEIS results for Farm-18BW. Manhattan plot showing the -log10 SNP-environment interaction p-values from the GWEIS. Gene labels have been annotated to suggestively significant (p < p <1/598,430 = 1.67e-6) independent SNPs with the lowest p-value.

Farm-Weight of 24 months (24BW)

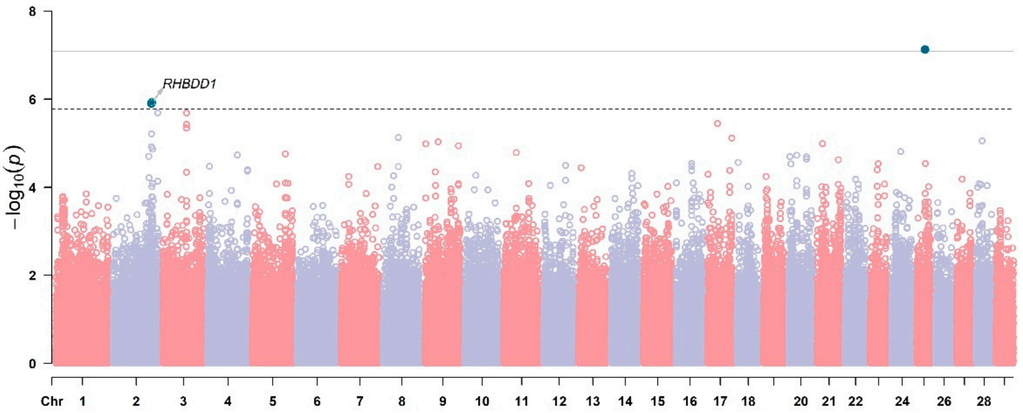

Supplementary Figure S4. Manhattan plot of GWEIS results for Farm-24BW. Manhattan plot

showing the -log10 SNP-environment interaction p-values from the GWEIS. Gene labels have been annotated to suggestively significant (p < p <1/598,430 = 1.67e-6) independent SNPs with the lowest p-value.

Temperature- Weaning weight (WW)

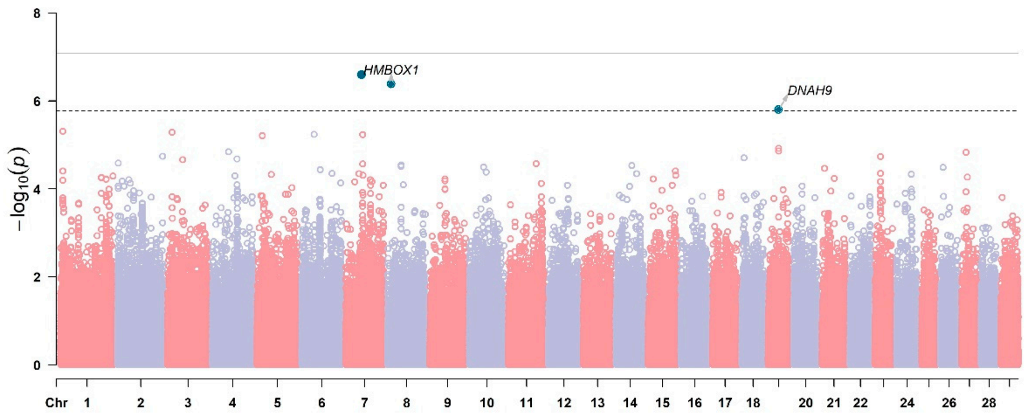

Supplementary Figure S5. Manhattan plot of GWEIS results for Temperature-WW. Manhattan plot showing the -log10 SNP-environment interaction p-values from the GWEIS. Gene labels have been annotated to suggestively significant (p < p <1/598,430 = 1.67e-6) independent SNPs with the lowest p-value.

Temperature-Yearling weight (YW)

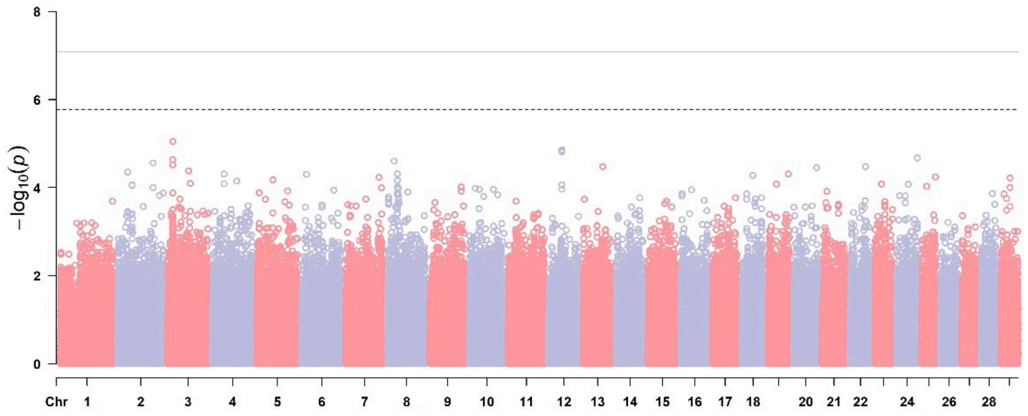

Supplementary Figure S6. Manhattan plot of GWEIS results for Temperature-YW. Manhattan plot showing the -log10 SNP-environment interaction p-values from the GWEIS. Gene

labels have been annotated to suggestively significant (p < p <1/598,430 = 1.67e-6) independent SNPs with the lowest p-value.

Temperature-Weight of 18 months (18BW)

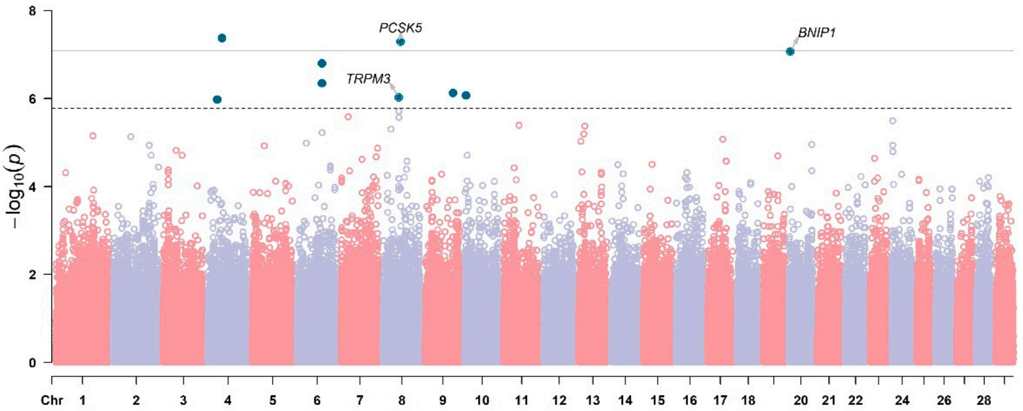

Supplementary Figure S7. Manhattan plot of GWEIS results for Temperature-18BW. Manhattan plot showing the -log10 SNP-environment interaction p-values from the GWEIS. Gene labels have been annotated to suggestively significant (p < p <1/598,430 = 1.67e-6) independent SNPs with the lowest p-value.

Temperature-Weight of 24 months (24BW)

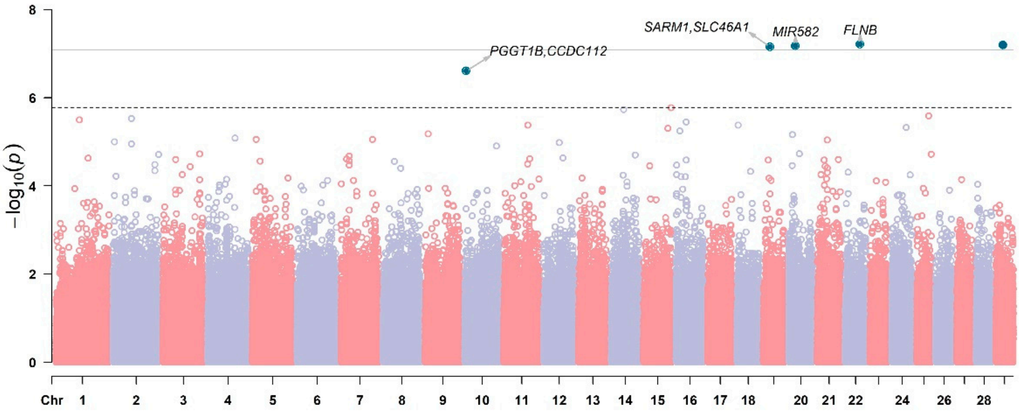

Supplementary Figure S8. Manhattan plot of GWEIS results for Temperature-24BW. Manhattan plot showing the -log10 SNP-environment interaction p-values from the GWEIS. Gene labels have been annotated to suggestively significant (p < p <1/598,430 = 1.67e-6) independent

SNPs with the lowest p-value.

Interaction Plots

These plots show the adjusted growth trait phenotypes at any given combination of alleles and environmental factor.

Farm-Weaning weight (WW)

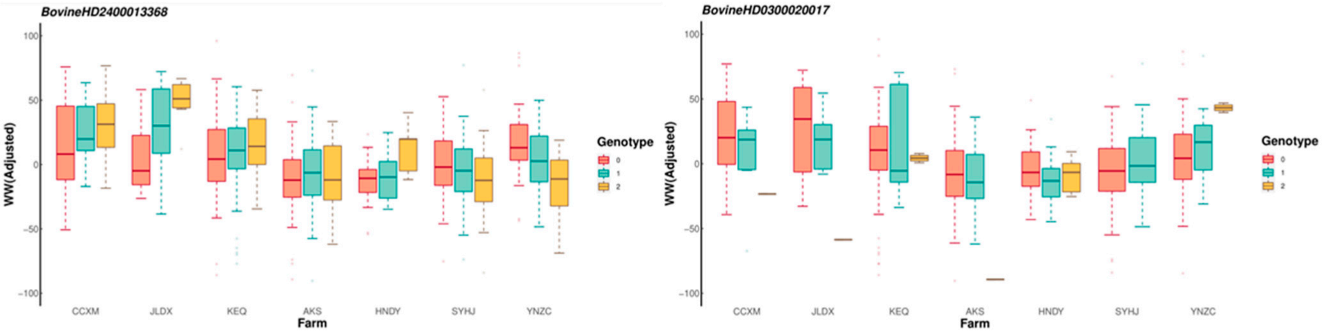

Supplementary Figure S9. Interaction plot of significant SNPs for Farm-WW. Interaction plots of adjusted weaning weight changing over farms for different genotypes of significant SNPs.

Farm-Yearling weight (YW)

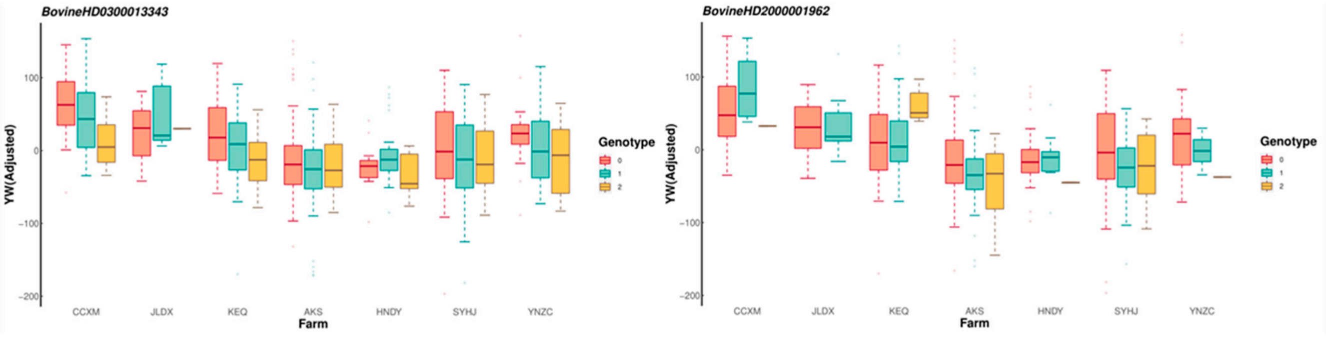

Supplementary Figure S10. Interaction plot of significant SNPs for Farm-YW. Interaction plots of adjusted yearling weight changing over farms for different genotypes of significant SNPs.

Farm-Weight of 18 months (18BW)

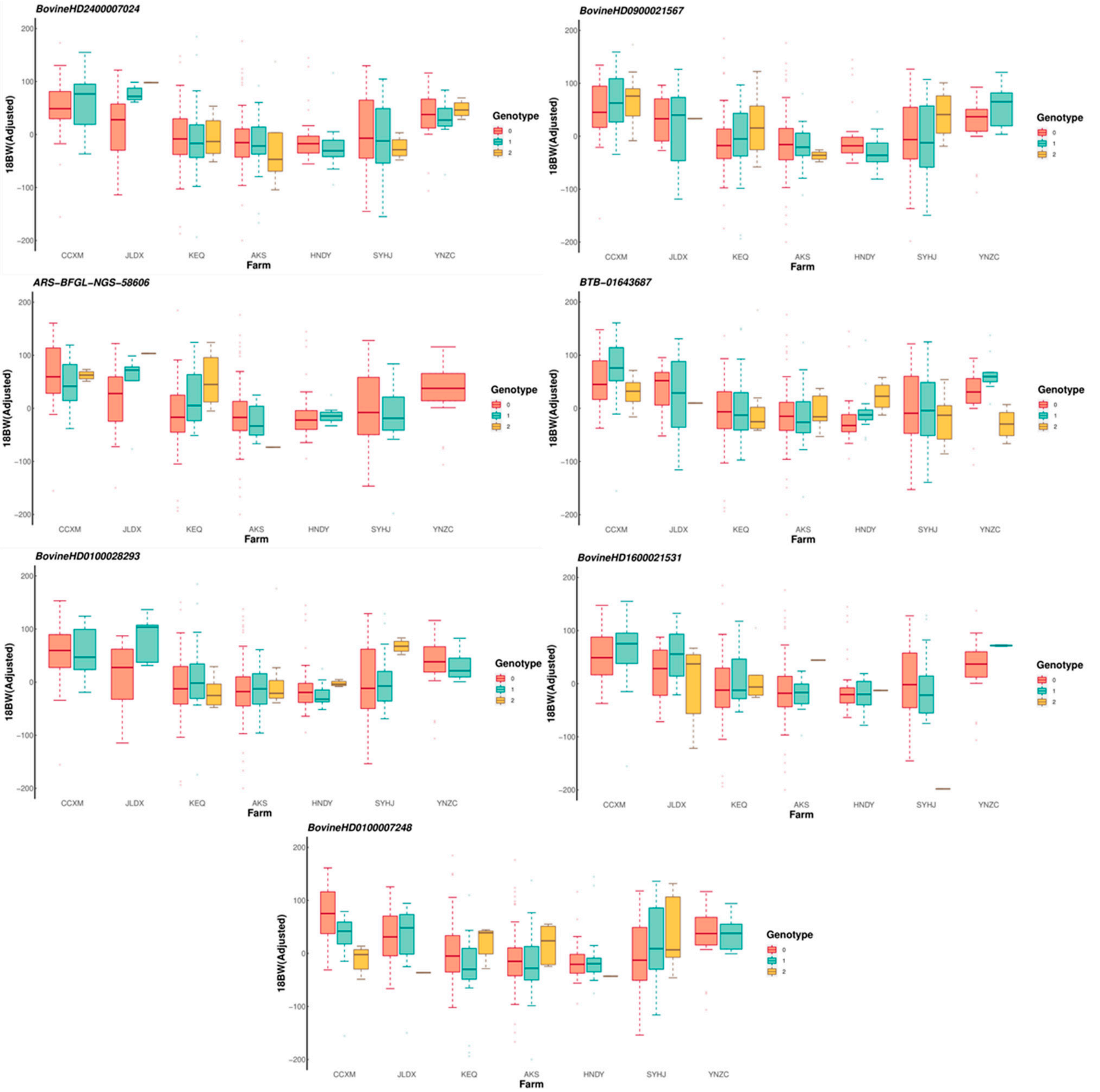

Supplementary Figure S11. Interaction plot of significant SNPs for Farm-18BW. Interaction plots of adjusted weight of 18 months changing over farms for different genotypes of significant SNPs.

Farm-Weight of 24 months (24BW)

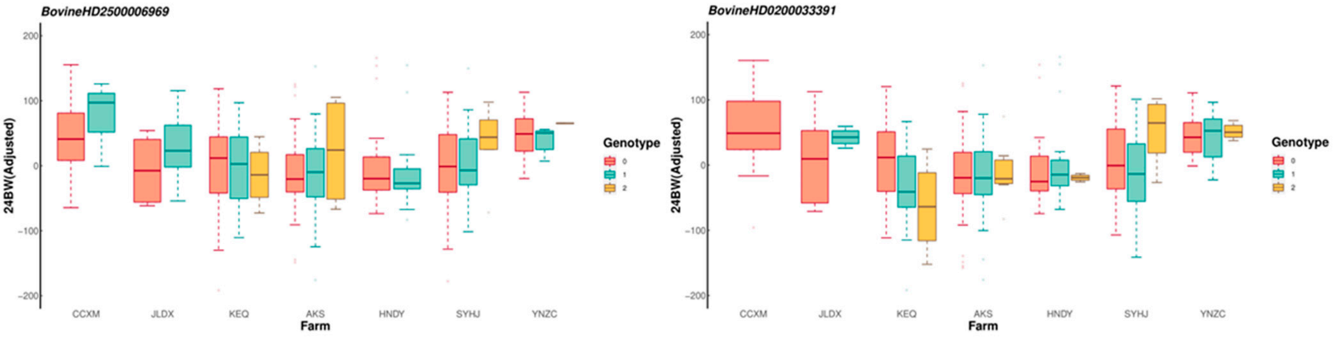

Supplementary Figure S12. Interaction plot of significant SNPs for Farm-24BW. Interaction plots of adjusted weight of 24 months changing over farms for different genotypes of significant SNPs.

Temperature- Weaning weight (WW)

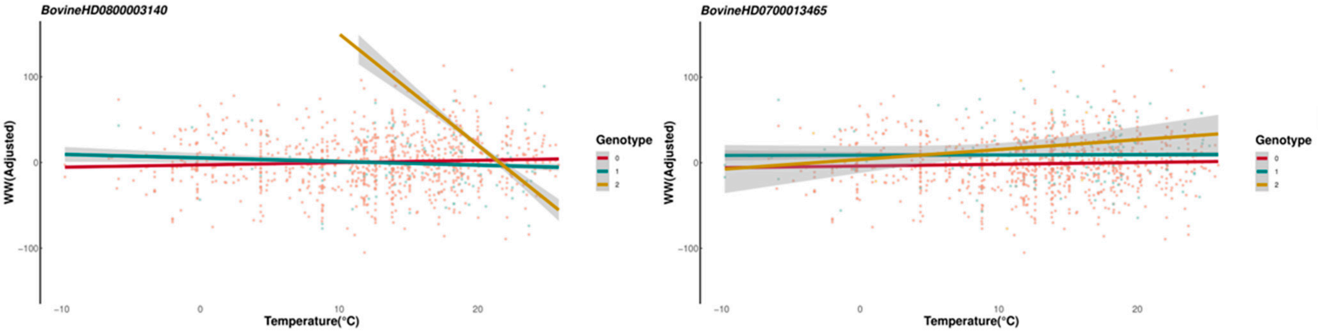

Supplementary Figure S13. Interaction plot of significant SNPs for Temperature-WW. Interaction plots of adjusted weaning weight changing over mean monthly temperature for different genotypes of significant SNPs.

Temperature-Weight of 18 months (18BW)

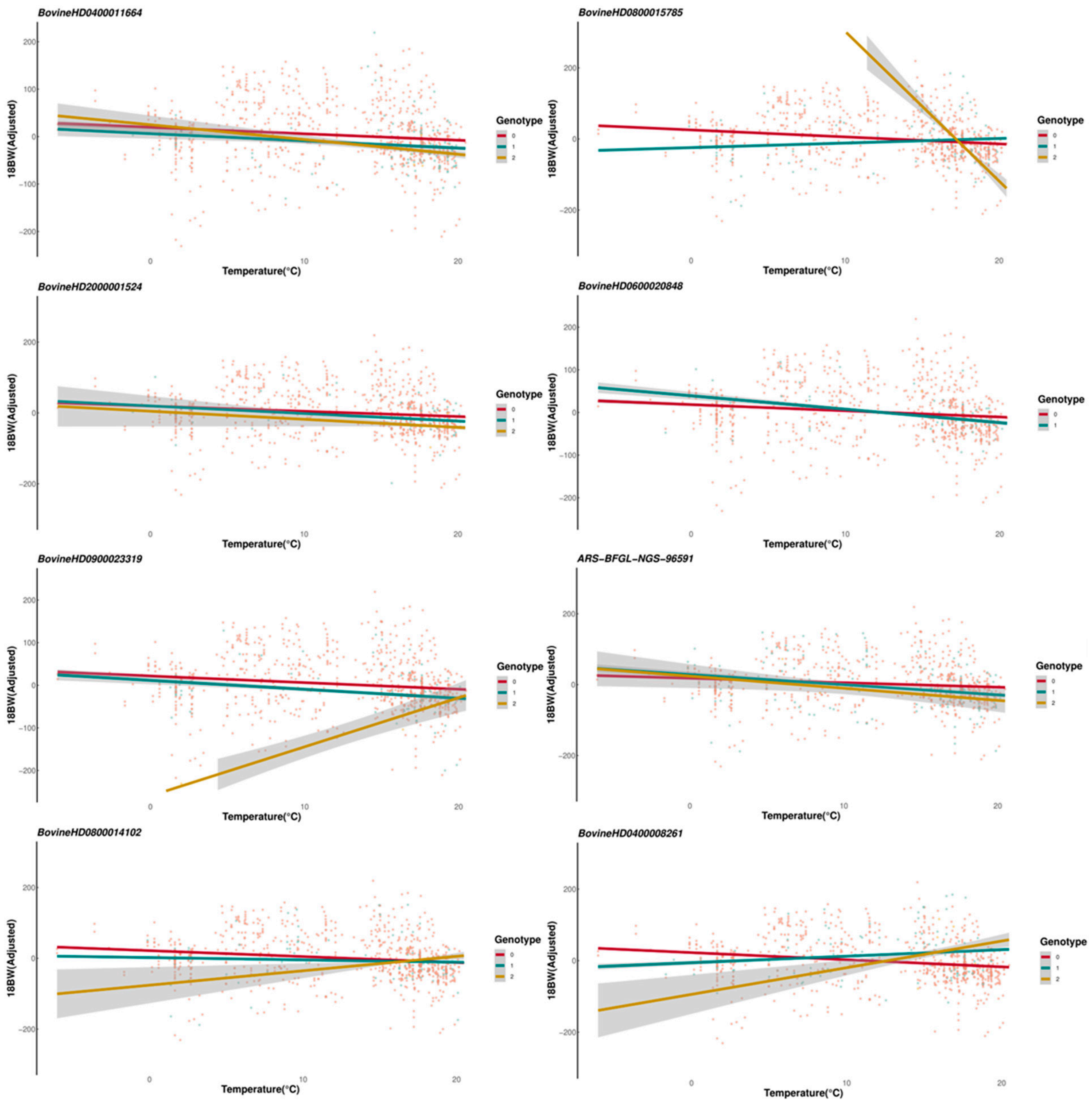

Supplementary Figure S14. Interaction plot of significant SNPs for Temperature-18BW. Interaction plots of adjusted weight of 18 months changing over mean monthly temperature for different genotypes of significant SNPs.

Temperature-Weight of 24 months (24BW)

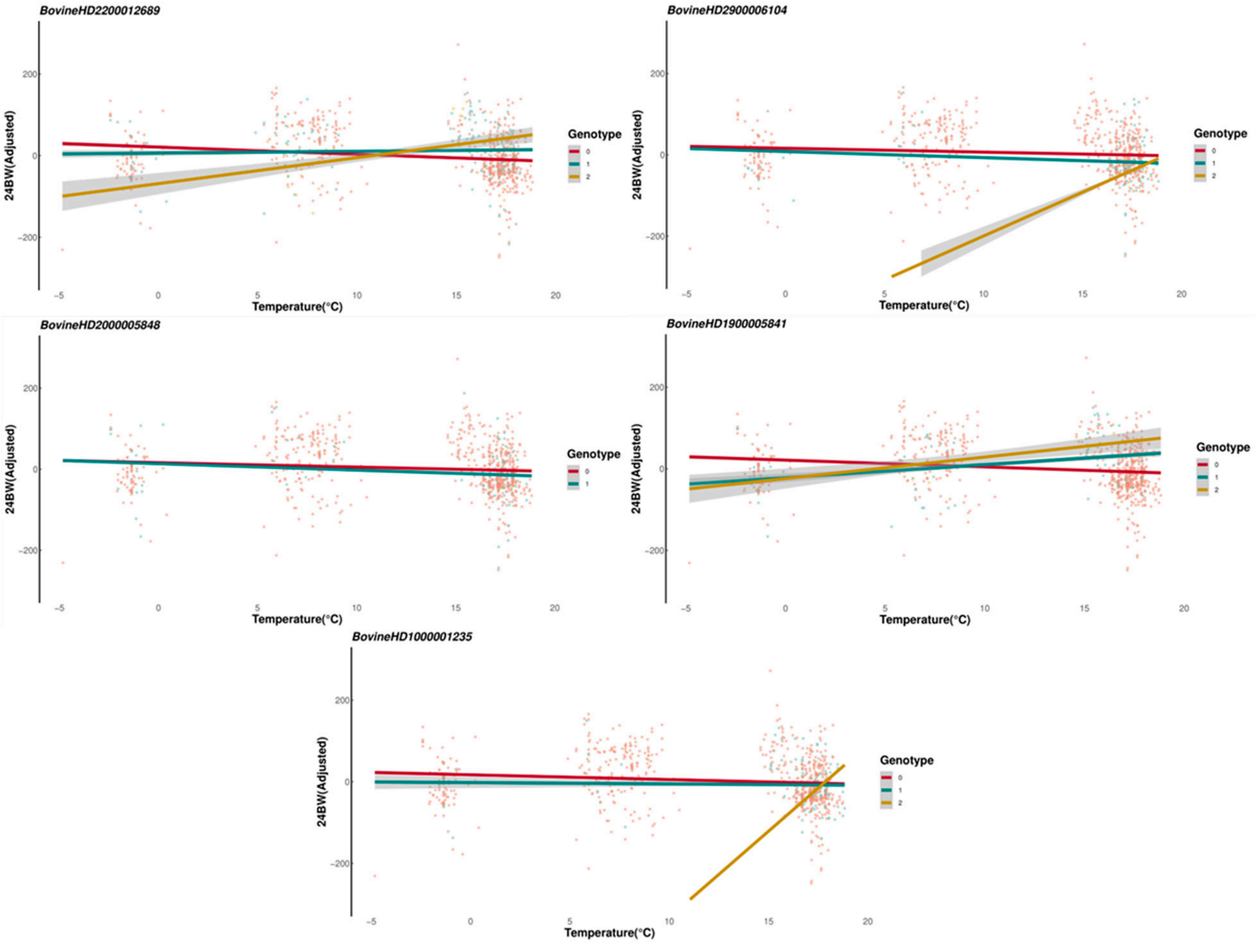

Supplementary Figure S15. Interaction plot of significant SNPs for Temperature-24BW. Interaction plots of adjusted weight of 24 months changing over mean monthly temperature for different genotypes of significant SNPs.

Gene Analysis Manhattan Plot

Shows the -log10 p-values for the MAGMA gene analysis (based on the GWEIS SNP-environment interaction results). Suggestively significant genes (p < 1e-4) have been annotated.

Farm-Weaning weight (WW)

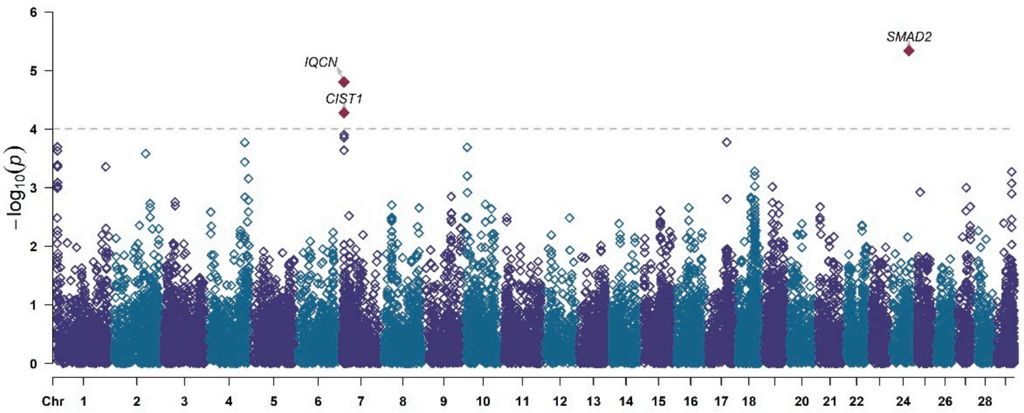

Supplementary Figure S16. Manhattan plot of MAGMA gene analysis results for Farm-WW. Manhattan plot showing the -log10 p-values for the MAGMA gene analysis. Suggestively significant genes (p < 1e-4) have been annotated.

Farm-Yearling weight (YW)

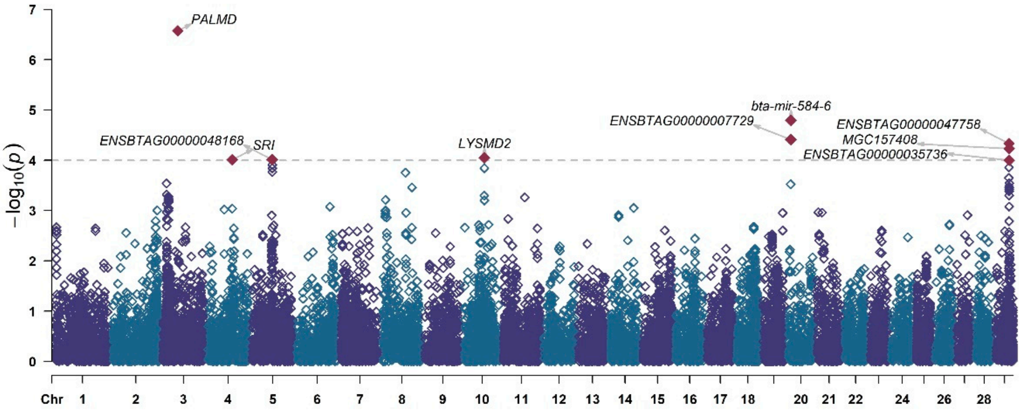

Supplementary Figure S17. Manhattan plot of MAGMA gene analysis results for Farm-YW.

Manhattan plot showing the -log10 p-values for the MAGMA gene analysis. Suggestively significant genes (p < 1e-4) have been annotated.

Farm-Weight of 18 months (18BW)

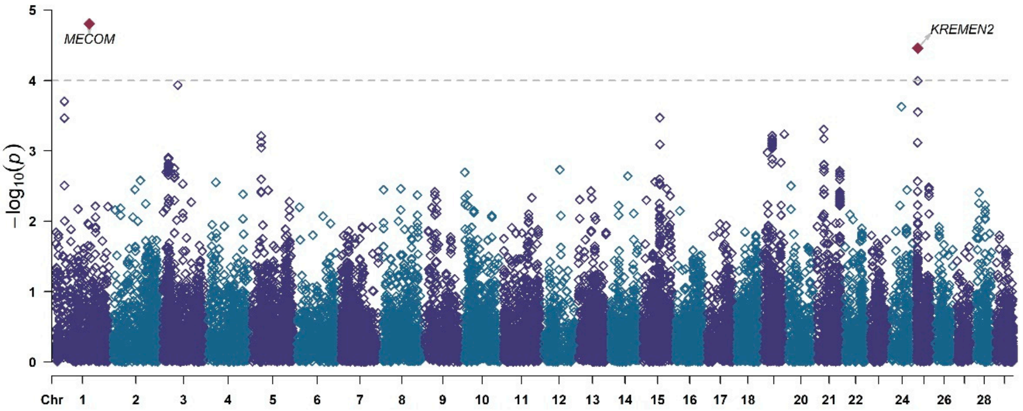

Supplementary Figure S18. Manhattan plot of MAGMA gene analysis results for Farm-18BW. Manhattan plot showing the -log10 p-values for the MAGMA gene analysis. Suggestively significant genes (p < 1e-4) have been annotated.

Farm-Weight of 24 months (24BW)

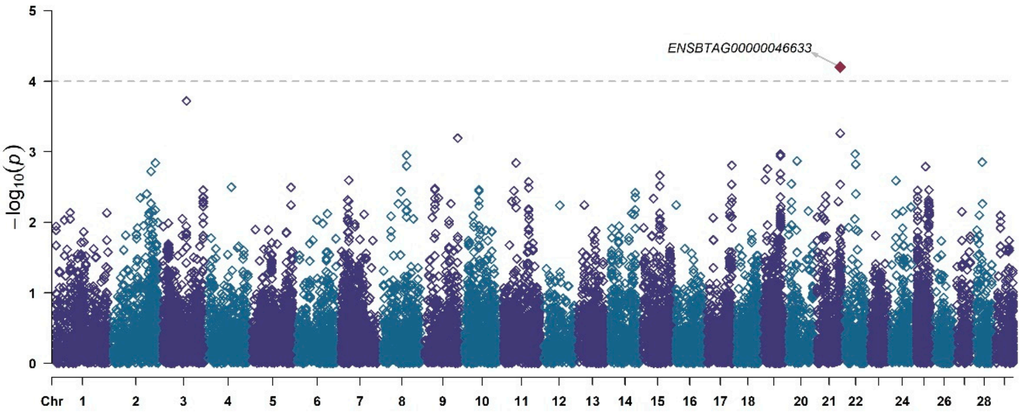

Supplementary Figure S19. Manhattan plot of MAGMA gene analysis results for Farm-24BW. Manhattan plot showing the -log10 p-values for the MAGMA gene analysis. Suggestively significant genes (p < 1e-4) have been annotated.

Temperature- Weaning weight (WW)

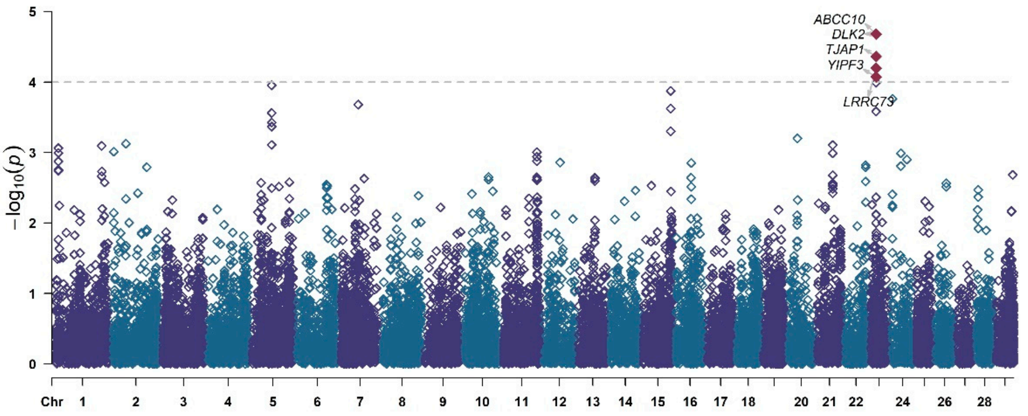

Supplementary Figure S20. Manhattan plot of MAGMA gene analysis results for temperature-WW. Manhattan plot showing the -log10 p-values for the MAGMA gene analysis. Suggestively significant genes (p < 1e-4) have been annotated.

Temperature-Yearling weight (YW)

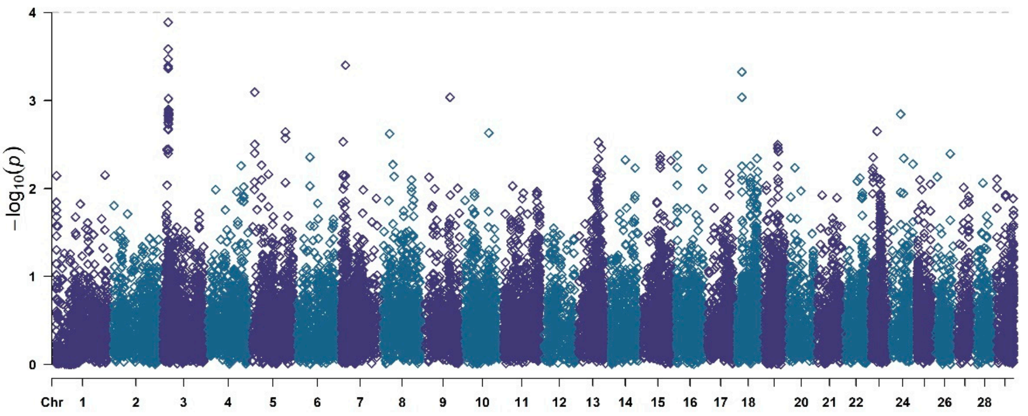

Supplementary Figure S21. Manhattan plot of MAGMA gene analysis results for temperature-YW. Manhattan plot showing the -log10 p-values for the MAGMA gene analysis. Suggestively significant genes (p < 1e-4) have been annotated.

Temperature-Weight of 18 months (18BW)

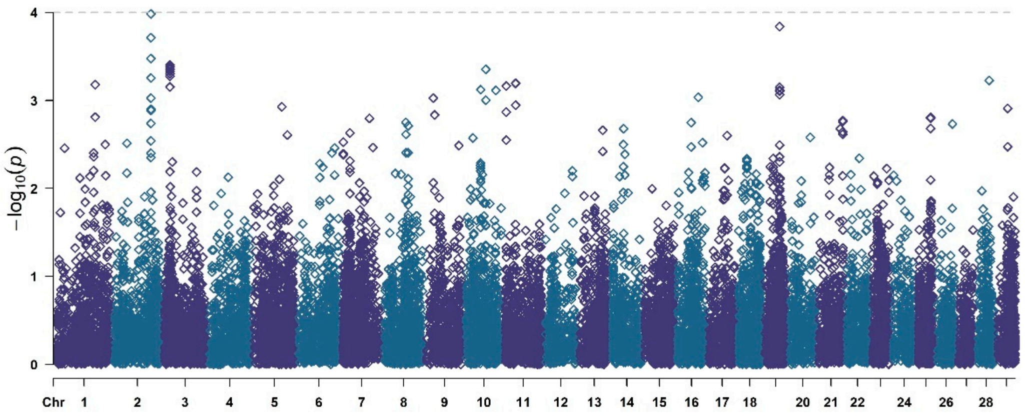

Supplementary Figure S22. Manhattan plot of MAGMA gene analysis results for temperature-18BW. Manhattan plot showing the -log10 p-values for the MAGMA gene analysis. Suggestively significant genes (p < 1e-4) have been annotated.

Temperature-Weight of 24 months (24BW)

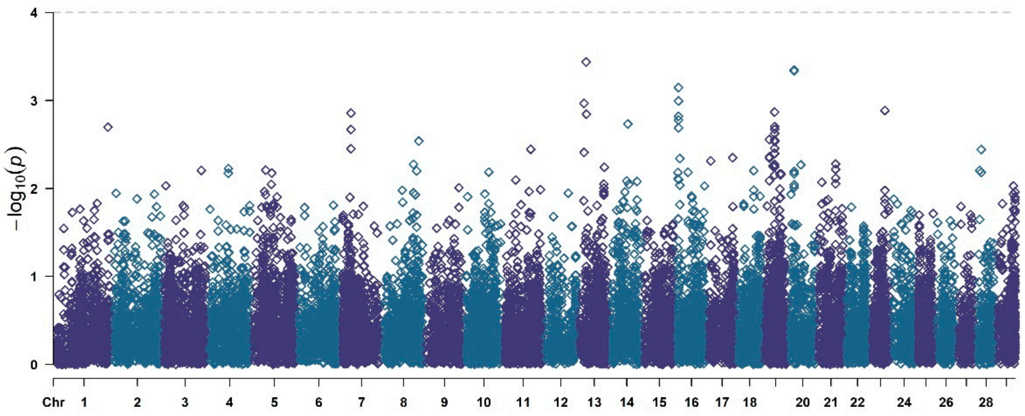

Supplementary Figure S23. Manhattan plot of MAGMA gene analysis results for temperature-24BW. Manhattan plot showing the -log10 p-values for the MAGMA gene analysis. Suggestively significant genes (p < 1e-4) have been annotated.
